# P-643. Incidence and Clinical Characteristics of Invasive Group A Streptococcal Infections in Underhoused Adults in Toronto, Canada

**DOI:** 10.1093/ofid/ofae631.840

**Published:** 2025-01-29

**Authors:** Caroline Kassee, Halima Dabaja Younis, Zoe Zhong, Vanessa Allen, Irene Armstrong, Mahin Baqi, Kevin R Barker, Ari Bitnun, Sergio Borgia, Aaron Campigotto, Sumon Chakrabarti, Wayne Gold, Alyssa Golden, Jennie Johnstone, Christopher Kandel, Ian Kitai, Julianne Kus, Liane Macdonald, Irene Martin, Matthew Muller, Jeya Nadarajah, Daniel R Ricciuto, David Richardson, Medina Saffie, Manal Tadros, Monali Varia, Kazi Hassan, Maxime Lefebvre, Angel Li, Shiva Barati, Gloria Crowl, Lubna Farooqi, Nadia Malik, Mare Pejkovska, Asfia Sultana, Tamara Vikulova, Allison McGeer

**Affiliations:** Sinai Health System, Toronto, Ontario, Canada; Sinai Health, Toronto, Ontario, Canada; Sinai Health System, University of Toronto, Toronto, Ontario, Canada; Sinai Health System, University of Toronto, Toronto, Ontario, Canada; Toronto Public Health, Toronto, Ontario, Canada; William Osler Health System, Toronto, Ontario, Canada; Trillium Health Partners, Toronto, Ontario, Canada; Hospital for Sick Children, Toronto, ON, Canada; William Osler Health System, Toronto, Ontario, Canada; Hospital for Sick Children, Toronto, ON, Canada; Trillium Health Partners, Toronto, Ontario, Canada; University of Toronto, Toronto, ON, Canada; University of Manitoba, Winnipeg, Manitoba, Canada; University of Toronto, Toronto, ON, Canada; Michael Garron Hospital, Toronto, Ontario, Canada; University of Toronto, Toronto, ON, Canada; Public Health Ontario, Toronto, Ontario, Canada; Public Health Ontario, Toronto, Ontario, Canada; National Microbiology Laboratory (NML), Winnipeg, MB, Canada; Unity Health, University of Toronto, Toronto, Ontario, Canada; Oak Valley Heatlh, Toronto, Ontario, Canada; Lakeridge Health, Oshawa, Ontario, Canada; William Osler Health System, Toronto, Ontario, Canada; Joseph Brant Hospital, Burlington, Ontario, Canada; The Hospital for Sick Children, Toronto, Ontario, Canada; Peel Public Health, Mississauga, Ontario, Canada; Sinai Health System, Toronto, Ontario, Canada; Sinai Health System, Toronto, Ontario, Canada; Sinai Health System, Toronto, Ontario, Canada; Sinai Health, Toronto, Ontario, Canada; Michael Garron Hospital, Toronto, Ontario, Canada; Sinai Health System, University of Toronto, Toronto, Ontario, Canada; Sinai Health, Toronto, Ontario, Canada; Sinai Health System, Toronto, Ontario, Canada; Sinai Health, Toronto, Ontario, Canada; Sinai Health, Toronto, Ontario, Canada; Mt. Sinai Hospital, Toronto, Ontario, Canada

## Abstract

**Background:**

Post-pandemic increases of invasive group A streptococcal disease (iGAS) have been reported in many areas of the world; increased risk associated with illicit intravenous (IV) drug use has also been identified.

**Figure:**
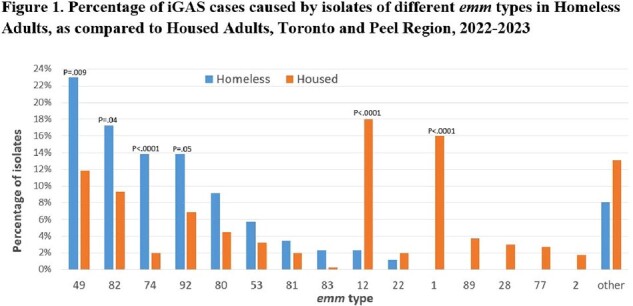

**Methods:**

Since 1995, the Toronto Invasive Bacterial Diseases Network (TIBDN) has conducted prospective, population-based surveillance for iGAS in residents of Toronto/Peel Region (pop, 4.5M) in Canada. Homeless adults admitted to hospitals in the surveillance area are counted as residents. General and homeless population estimates were taken from Statistics Canada and published literature (doi:10.1136/bmjopen-2019-030221). Canada's National Microbiology Laboratory performs *emm* typing.

**Figure:**
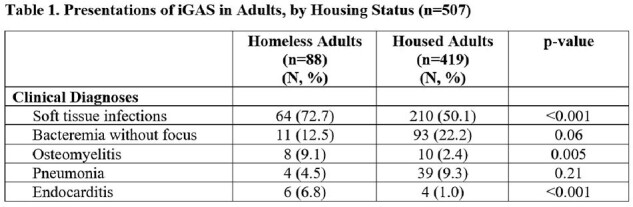

**Results:**

In 2022/23, 88 iGAS cases (14% of all iGAS cases, 17% of adult iGAS) occurred in homeless persons; all were adults. Estimated iGAS incidence in homeless adults was 210/100,000/yr, versus 5.4/100,000/yr in other adults. Homeless persons with iGAS were younger than other adults (median age 47 v. 59 yr, P< .001); 70% were male (v. 63% in other adults, P=0.2). Homeless adults were more likely than other adults to be smokers (53% v. 27%, P< .001), to use IV drugs (46% v. 10%, P< .001), to have non-intact skin (43% v. 27%, P< .001) and to report recent soft tissue trauma (27% v. 17 %, P< .001). Compared to other adults, homeless adults were more likely to have comorbidities (90% v. 79%, P< .001), but less likely to be immunocompromised (3% v. 19%, P< .001). Presentations differed in homeless versus other adults (Table 1). 16 (18%) homeless adults required ICU admission, 10 (11%) mechanical ventilation, and 5 (8.5%) died. After age adjustment, outcomes did not differ between homeless and other adults. The median hospital length of stay was 8.5 days (IQR: 4-22) for homeless adults, and 8 days (IQR: 5-18) for other adults (P=0.7). The distribution of *emm* types of isolates in homeless and other adults is shown in Figure 1. iGAS in homeless adults was less likely to be caused by isolates of *emm* 1 and 12, and more likely to be caused by isolates of *emm* 49 and 74.

**Conclusion:**

Homeless persons are at very high risk of iGAS, with IV drug use and skin integrity issues contributing. Outcomes of iGAS are similar to other adults. Differences in the *emm* types of isolates may be relevant to future vaccine program planning.

**Disclosures:**

**Allison McGeer, MD**, AstraZeneca: Honoraria|GSK: Honoraria|Merck: Honoraria|Moderna: Honoraria|Novavax: Honoraria|Pfizer: Grant/Research Support|Pfizer: Honoraria|Roche: Honoraria|Seqirus: Grant/Research Support|Seqirus: Honoraria

